# Cognitive Dysfunction in Early Multiple Sclerosis: Altered Centrality Derived from Resting-State Functional Connectivity Using Magneto-Encephalography

**DOI:** 10.1371/journal.pone.0042087

**Published:** 2012-07-27

**Authors:** Martin Hardmeier, Menno M. Schoonheim, Jeroen J. G. Geurts, Arjan Hillebrand, Chris H. Polman, Frederik Barkhof, Cornelis J. Stam

**Affiliations:** 1 Department of Clinical Neurophysiology and Magnetoencephalography Center, VU University Medical Center, Amsterdam, The Netherlands; 2 Department of Neurology, Division of Clinical Neurophysiology, Hospital of the University of Basel, Basel, Switzerland; 3 Department of Radiology, VU University Medical Center, Amsterdam, The Netherlands; 4 Department of Anatomy and Neuroscience (section of Clinical Neuroscience), VU University Medical Center, Amsterdam, The Netherlands; 5 Department of Neurology, VU University Medical Center, Amsterdam, The Netherlands; Cuban Neuroscience Center, Cuba

## Abstract

**Background:**

Cognitive dysfunction in multiple sclerosis (MS) is frequent. Insight into underlying mechanisms would help to develop therapeutic strategies.

**Objective:**

To explore the relationship of cognitive performance to patterns of nodal centrality derived from magneto-encephalography (MEG).

**Methods:**

34 early relapsing-remitting MS patients (median EDSS 2.0) and 28 age- and gender-matched healthy controls (HC) had a MEG, a neuropsychological assessment and structural MRI. Resting-state functional connectivity was determined by the synchronization likelihood. Eigenvector Centrality (EC) was used to quantify for each sensor its connectivity and importance within the network. A cognition-score was calculated, and normalized grey and white matter volumes were determined. EC was compared per sensor and frequency band between groups using permutation testing, and related to cognition.

**Results:**

Patients had lower grey and white matter volumes than HC, male patients lower cognitive performance than female patients. In HC, EC distribution showed highest nodal centrality over bi-parietal sensors (“hubs”). In patients, nodal centrality was even higher bi-parietally (theta-band) but markedly lower left temporally (upper alpha- and beta-band). Lower cognitive performance correlated to decreased nodal centrality over left temporal (lower alpha-band) and right temporal (beta-band) sensors, and to increased nodal centrality over right parieto-temporal sensors (beta-band). Network changes were most pronounced in male patients.

**Conclusions:**

Partial functional disconnection of the temporal regions was associated with cognitive dysfunction in MS; increased centrality in parietal hubs may reflect a shift from temporal to possibly less efficient parietal processing. To better understand patterns and dynamics of these network changes, longitudinal studies are warranted, also addressing the influence of gender.

## Introduction

Cognitive dysfunction affects between 30 and 70% of patients with Multiple Sclerosis (MS) [1] and is a negative predictor of psycho-social functioning [2]. The most commonly identified impaired cognitive domains are attention, speed of information processing and memory [3].

Gaining deeper insight into the mechanisms of cognitive decline would help to develop therapeutic strategies. So far, it has been hypothesized from task-related functional Magnetic Resonance Imaging (fMRI) that increased activation of normally activated and activation of additional regions in MS patients reflect adaptive changes to structural disconnection [4]–[7]. In contrast, higher functional connectivity in the default mode as well as attention and cognitive control network correlated to poorer cognitive performance in early MS in a recent resting-state fMRI study [8], which seems to contradict a straightforward compensation hypothesis.

Electro- and magneto-encephalography (EEG and MEG) are tools to measure brain function directly with high temporal resolution. Synchronization of brain oscillations between different regions most likely reflects functional interaction as has been inferred from task specific synchronization changes [9]–[11]. Similarly, functional interaction is very likely to take place in the resting-state. As cognition results from dynamic interaction between distributed brain areas [12], a network perspective is suitable for gaining insight into brain functioning [13]–[14] and changes due to disease [15].

The synchronization likelihood (SL) is a measure to quantify functional connectivity in electrophysiological time series and accounts for linear and non-linear inter-relations [16]. SL has shown altered resting-state functional connectivity in Alzheimer’s disease (AD) [17], Parkinson’s disease [18], glioma patients with epilepsy [19] and MS [20].

In a network approach, each MEG sensor can be viewed as a node and each SL value between two sensors as the strength or “weight” of the link between two nodes [13], [21]. Topographic patterns of networks can be characterized with a range of measures [21] among which centrality measures quantify the importance of single nodes within the network [22]–[24]. Eigenvector Centrality (EC) weighs the connections of a node [25]–[26]: being connected to a highly connected “hub” makes a node more influential than being connected to many poorly connected peripheral nodes. In this way, EC takes the relation within the whole network into account and allows for the identification of hubs: these may play a crucial role in the development of cognitive symptoms as suggested from studies in AD [27]–[28].

Connectivity studies based on EEG or MEG in MS are scarce. In progressive patients, Leocani et al. [29] found decreased inter-hemispheric (theta-band) and intra-hemispheric (alpha-band) coherence using EEG, more pronounced in the cognitively impaired subgroup. Cover et al. [30] observed decreased inter-hemispheric connectivity (alpha-band), most pronounced in temporal regions using MEG. Schoonheim et al. [20] reported in a previous SL-based analysis of the same dataset as used in the present study higher inter- and intra-hemispheric connectivity originating mostly in parietal and occipital areas (theta-, lower alpha- and beta-band) and decreased inter-hemispheric connectivity between temporal regions (upper alpha-band) as well as a strong gender effect to the disadvantage of male patients.

In the present study, we extended our previous analysis to explore patterns of nodal centrality and their relation to cognitive performance by using EC. We expected this approach to be more sensitive to disease related changes, as EC quantifies not only local connectivity but also the importance of a node within the whole network, and moreover is a normalized measure. In contrast, SL and other measures of functional connectivity give only absolute numbers or weights of connections. We will discuss the implications of our results with regard to possible mechanisms of cognitive dysfunction.

## Methods

### Participants

Thirty-four MS patients (17 women, mean age 41.4+/−8 years, mean disease duration (8.1+/−1.6 years based on first symptom) from an early inception cohort (five to seven years after diagnosis) were studied. All patients had clinically definite MS [31], and a relapsing-remitting disease course (RRMS). Twenty eight healthy subjects (14 women), matched for age, gender and duration of education served as a control group. None of the healthy controls suffered from any neurological or psychiatric disease, nor used any medication. The study protocol was approved by the Medical Ethical Committee of the VU University Medical Center. All subjects gave written informed consent before participation. A previous analysis has been performed on the same dataset [20], and on the subsample of healthy controls [32].

### Neuropsychological Evaluation

Details of the standardized neuropsychological examination are described in Schoonheim et al. [20]. In short, all participants were tested with the brief repeatable battery of neuropsychological tests (BRB-N) [33], comprising the selective reminding test (SRT), the 10/36 spatial recall test (SPART), the symbol digit modalities test (SDMT), the paced auditory serial addition test (PASAT) and the word list generation test (WLG). In addition, the Stroop color-word test, the concept shifting test (CST) and the memory comparison test (MCT) were administered [34]. Individual subjects’ test scores were converted to z-scores, using the means and standard deviations of the entire group of healthy subjects, and summarized into the following cognitive domains: information processing speed (SDMT), psychomotor functioning (CST, SDMT), attention (Stroop), verbal memory (SRT), working memory (MCT), executive functioning (CST, WLG) and visuo-spatial memory (SPART). Post hoc, the PASAT was excluded from analysis, as all patients had repeatedly performed the test in the past and showed significant learning effects compared to controls.

To capture the heterogeneity of cognitive dysfunction in MS in one number, a cognition-score was calculated by averaging the z-scores over the seven cognitive domains, mainly reflecting the overall cognitive capacity, rather than specific functions. Spearman’s rho between the cognition-score and information processing speed, psychomotor functioning, attention, verbal memory, working memory, executive functioning and visuo-spatial memory was 0.93, 0.93, 0.54, 0.68, 0.39, 0.79 and 0.65, respectively.

### Magnetoencephalography

Details of the MEG recording and selection of epochs for further analysis have been described in [32]. Shortly, a 151-channel whole-head MEG system (CTF Systems Inc., Port Coquitlam, BC, Canada) inside a magnetically shielded room (Vacuumschmelze GmbH, Hanau, Germany) was used to record magnetic fields during a 5 minute eyes-closed resting period at a sampling frequency of 625 Hz. A third order software gradient was used after online band-pass filtering between 0.25 and 125 Hz. For each subject, five artefact-free epochs of 4096 samples (6.554 s) were selected. Epochs were band-pass filtered into the commonly used frequency bands: delta (0.5–4 Hz), theta (4–8 Hz), lower alpha (8–10 Hz), upper alpha (10–13 Hz), beta (13–30 Hz) and gamma (30–48 Hz). All further analyses were performed separately for these bands. Fourteen channels had to be excluded due to artefacts in one or more participants, leaving 137 channels for further analyses.

### Synchronization Likelihood

Synchronization likelihood (SL, see [16]) was used as an index of functional connectivity and was calculated with BrainWave (version 0.8.99; available from: http://home.kpn.nl/stam7883/brainwave.html). Shortly, SL measures the statistical interdependency of two time series by determining the probability that recurrent patterns (whatever their shape) in each time series occur at the same time, thus taking linear and non-linear relations into account. SL is based on the concept of generalized synchronization [35], and is more sensitive than linear measures like coherence or cross-correlation (for review see [36]). In the present study SL between all combinations of the 137 included channels was determined per frequency band (for specific parameter settings see [37]). In the resulting 137×137 connectivity matrices, each SL value represents the strength of a connection. In this way, the connectivity matrices are identical to weight matrices, which were subsequently used for the EC analysis. Weighted analysis avoids arbitrarily setting a threshold for binarization and results in fully connected graphs. Furthermore, it has been shown to more comprehensively characterize the network [38]–[39].

### Eigenvector Centrality

Centrality measures originate in the social sciences and determine which member of a network is the most influential [25], [40]. They have recently been introduced into neuroscience [22]–[24] and allow the determination of connectivity from the perspective of a single node, quantifying the importance of a node within a network. Degree centrality simply equals the number of connections of a node, its degree, or, in weighted networks the strengths of a node (the sum of the weights of all the edges of a node) [40]. In contrast, EC determines the relative importance of a node within the entire network by also considering the quality of the connections [25]–[26]. Furthermore, it is a vector-normalized measure facilitating comparisons between equal sized networks. EC will also be referred to as nodal centrality.

EC is based on the spectral decomposition of a binarized adjacency or a weight matrix. For symmetric matrices with strictly positive entries, the decomposition yields a unique largest real Eigenvector with strictly positive entries (Perron-Frobenius-theorem). This holds also for irreducible square matrices with non-negative entries, which is the case for the weight matrix derived from SL used in the present study. We calculated the EC according to Lohmann et al. [22] using BrainWave (version 0.8.99): The EC of a node *i* is defined as the *i*-th entry in the normalized Eigenvector belonging to the largest Eigenvalue.

Nodes with outstanding centrality can be viewed as the “hubs” of a network [21], [41]: sensors belonging in at least 80% of healthy subjects to the 20% highest EC-values of each individual subject were defined as hubs on a group level [42].

### Magnetic Resonance Imaging

Details of the MRI procedures and analyses are described in Schoonheim et al. [20]. In short, all subjects underwent an MRI scan using a 3T-MR-system (GE Signa HDXT, V15M), except two patients who refused scanning due to claustrophobia. A 3D-T1 FSPGR-, a 2D dual echo PD/T2- and a 2D spin echo T1-sequence were acquired. T1-hypointense and T2-hyperintense lesion volumes were quantified using Alice (Perceptive Informatics Inc.) and total grey and total white matter volumes corrected for head size were measured using SIENAX, version 2.5 [43].

### Statistical Analysis

As previous analyses showed gender differences in delta-band functional connectivity for healthy controls [32], and most pronounced group differences in male MS patients [20], group (MS vs. HC) and subgroup-comparisons (MS men vs. HC men, and MS women vs. HC women) were performed for the EC data. Clinical and MRI variables were compared between groups by a MANOVA, and within patients between gender (male vs. female MS patients) by a one-factor ANOVA.

For each frequency band, significance of EC differences between groups and subgroups was estimated by permutation testing, using maximum statistics in order to correct for multiple comparisons over sensors [44]. In permutation testing the null distribution for between-group differences is derived from the data: assuming no group differences, group assignment is permutated. In our setting a t-statistic was calculated after each permutation. To correct for multiple comparisons, the maximum t-value across sensors for each permutation was used to construct a distribution of maximum t-values for 5000 permutations. The threshold for alpha = 0.05 for this distribution of maximum values was calculated and subsequently applied to determine whether observed t-values at the individual sensors reached significance. As only differences between MS men and HC men survived this strict correction, uncorrected p-values are reported at a level of p<0.01.

To explore the relation between the EC and cognitive performance the normally distributed centrality values at each sensor were correlated to the cognition-score within the MS-group and within healthy controls by Pearson’s product moment correlation. To adjust for multiple comparisons, the false discovery rate was used [45]–[46].

The sensor-level results were visualized by plotting 1) z-maps to depict the distribution of EC-values in healthy controls after averaging per sensor and z-transformation, 2) t-maps to show the topographic patterns of group- and subgroup differences, and 3) r-maps to picture the topography of correlations between EC-values and the cognition-score separately for MS-patients and healthy controls.

## Results

### Clinical, Neuropsychological and MRI Measures

Table 1 summarizes clinical and MRI characteristics for healthy controls and MS patients, as well as male and female patients. A MANOVA with group and gender as fixed factors showed significantly lower grey and white matter volumes in MS patients (p<0.05 and p<0.01, respectively), a significant gender difference with lower grey matter volumes (p<0.01) in men, and a significant group by gender interaction for cognitive performance (p<0.05), where male patients had the lowest scores. Six male and two female MS patients performed below two standard deviations of healthy controls in at least one cognitive domain, signifying mild cognitive impairment. Within patients a one-factor ANOVA between gender showed significantly lower cognition-scores (p<0.05) and lower grey matter volume (p<0.01) in male patients.

**Table 1:** Baseline characteristics of healthy controls and MS patients.

	healthy controls	MS	MS women	MS men
n	28	34	17	17
age (y)	39.8 (10.5)	41.4 (8.0)	41.4 (5.7)	41.3 (10.0)
EDSS	NA	2.0 (0–4.5)	2.0 (0–4.5)	2.0 (0–4.0)
disease duration (y)	NA	8.1 (1.6)	8.32 (2.0)	7.86 (0.93)
“cognition” (z-value)	0.070 (0.61)	−0.278 (0.844)	0.091 (0.58)	**−0.647 (0.92)** +
NGMV (ml)	845.9 (49.8)	**815.4 (42.3)** *	833.3 (38.4)	**795.1 (38.1)^++^**
NWMV (ml)	691.7 (35.8)	662.9 (29.8)**	660.9 (25.2)	665.1 (35.0)
T2 (ml)	NA	1.50 (0.21–13.96)	1.18 (0.33–5.53)	2.26 (0.21–13.96)
T1 (ml)	NA	0.76 (0.04–9.23)	0.57 (0.13–3.87)	0.86 (0.04–9.23)

Means (±SD) are given, for EDSS, T2 and T1 medians (range); EDSS: expanded disability status scale; “cognition”: average of z-scores over seven cognitive domains; NGMV: normalized grey matter volume; NWMV: normalized white matter volume; T2: T2-hyperintense lesion volume; T1: T1-hypointense lesion volume.

* = p<0.05 and ** = p<0.01 for comparison between healthy controls and MS patients.

+ = p<0.05 and ^++^ = p<0.01 for comparison between MS women and MS men.

### Topography of Eigenvector Centrality in Healthy Controls

**Figure 1:**
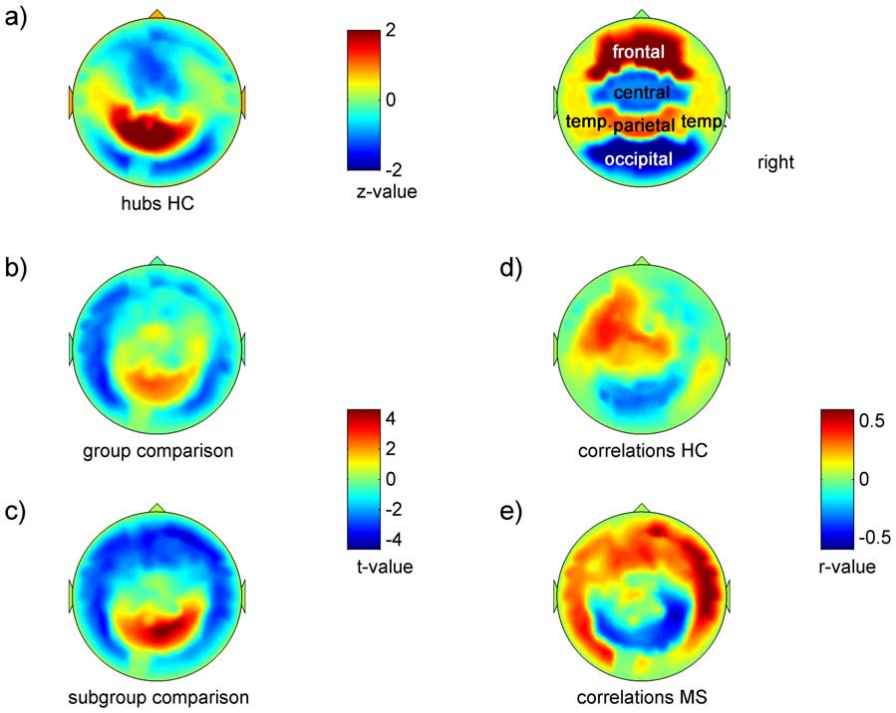
EC-distribution, group- and subgroup-comparisons and correlations of EC to “cognition” in the beta-band (13–30 Hz). Regions are plotted in the right upper corner; a) spatial distribution of z-transformed group averaged EC-values per sensor in healthy controls plotted as a z-map; b) group (MS vs. HC) and c) subgroup differences (MS men vs. HC men) plotted as t-maps; warm colors indicate higher values in MS; d) correlations between “cognition” and EC-value per sensor in healthy controls plotted as a r-map, warm colors indicate positive correlation; e) same as d) in MS-patients.

**Figure 2:**
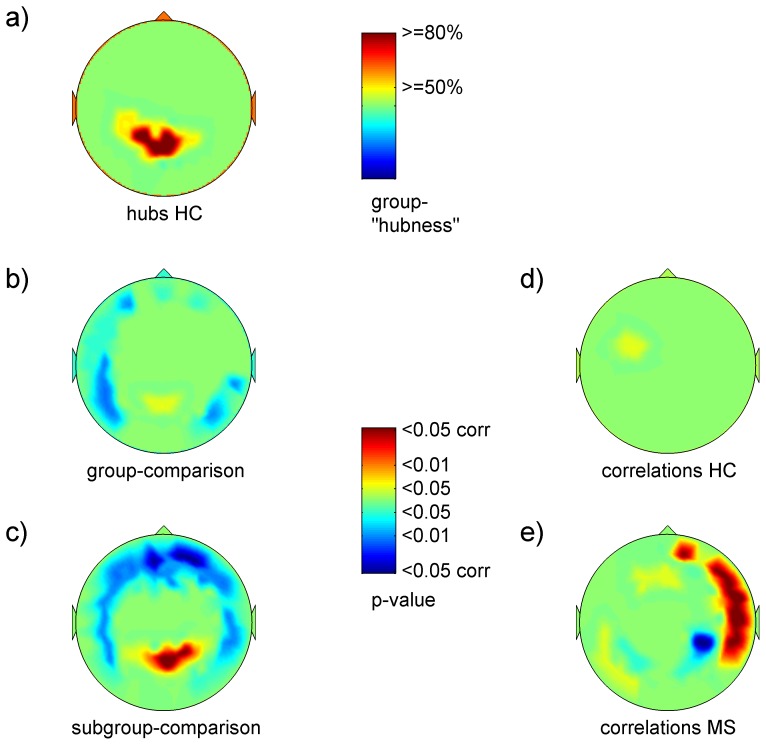
Hubs and p-maps corresponding to Fig. 1: a) spatial distribution of sensors, which belong in > = 50% and > = 80% of healthy subjects to the 20% highest ranks (group-“hubness”) corresponding to **Figure 1a**); b)–e) p-values for corresponding t- and r-maps of **Figure 1b–e**.

Figures 1 and 2 give a synopsis of the different analyses in the beta-band, which showed the most prominent changes; Figures S1, S2, S3, S4, S5 show the analyses over each of the six frequency bands.

Figure 1a and S1 depict the spatial distribution of EC-values in healthy controls as z-maps. As there were no significant (p<0.01) gender differences, healthy subjects are described as one group. In the delta-band (Figure S1a), EC-values were highest over the right fronto-temporal region, and medium high values were found over the left fronto-temporal region. In contrast, from the theta- to the gamma-band the pattern of distribution showed highest EC-values over both parietal areas with a clear preponderance of the left side, and medium high EC-values over both temporal regions also with a left dominance (Figure S1b–f). This pattern was most apparent in the gamma-band. Furthermore, inter-individual variability was lower in the higher frequency bands (upper alpha- to gamma-band), indicated by the higher number of sensors in which highest individual EC-values were at the same sensors in over 80% of subjects. These hubs were located over the left temporo-parietal junction in the upper alpha- and gamma-band, and bi-parietally in the beta-band (Figure 2a and S1d–f).

### Group and Subgroup Differences in Topography of Eigenvector Centrality

Table 2 gives the number of significantly different sensors per region for group and sub-group comparisons, and Figures 1b, 2b and S2 show the distribution of group differences in EC-values as t-maps and corresponding p-values. In the delta- to beta-band the pattern of group differences was similar: MS patients had bi-parietally higher EC-values (theta-band, p<0.01, Figure S2b), and lower EC-values mainly over left temporal regions (upper alpha and beta-band, p<0.01, Figure S2d–e). The gamma-band showed a different pattern: here MS-patients had lower EC-values over right parietal regions (p<0.01, Figure S2f).

**Table 2:** Group and subgroup comparisons: number of significantly different sensors per region, side and frequency band.

	frontal		temporal		central		Parietal		occipital	
band	left	right	left	right	left	right	left	right	left	right
delta	–	–	–	**1**/2	–	−/1	−/2	**1**/3(2)	–	−/2
theta	–	–	–	–	–	**1**/−	**1**/1(1)	**2**/2	**1**/1	–
lower alpha	−/1	–	**1**/4(1)	−/4	–	–	−/2	−/3	−/1	–
upper alpha	–	−/1	**7**/3	**3**/8	**1**/−	–	−/2	–	–	–
beta	**1**/9(1)	−/9(2)	**6**/7	**1**/8	–	–	−/2(1)	−/3(2)	**1**/1	**1**/2(1)
gamma	–	–	–	–	–	–	–	**3**/−	–	–

Number of sensors with significant differences (p<0.01; in brackets: p<0.05 corrected) between groups (bold) and subgroups (male MS patients vs. healthy men, standard font). Frequency bands: delta: 0.5–4.0 Hz, theta: 4.0–8.0 Hz, lower alpha: 8.0–10.0 Hz, upper alpha: 10.0–13.0 Hz, beta: 13.0–30.0 Hz, gamma: 30.0–48.0 Hz.

Figures 1c, 2c and S3 show the distribution of subgroup differences (male MS patients vs. healthy men). The patterns were quite similar to the patterns of the group comparisons, but were more pronounced and additionally more widespread in the beta-band,: EC-values were significantly higher in bi-parietal sensors (p<0.01, delta- to beta-band; p<0.05 corrected in delta-, theta- and beta-band; Figures 2c and S3a–e) and significantly lower in temporal sensors (p<0.01, delta-, lower alpha to beta-band; p<0.05 corrected, lower alpha-band, Figures 2c and S3a, c–e). Additionally, bi-frontal sensors showed significantly lower EC-values in males with MS (p<0.01 and p<0.05 corrected; beta-band; Figures 1c and S3e). In the gamma-band, there were no significant differences between the male subgroups (Figure S3f). Female patients did not show significant differences at p<0.01 to healthy women.

Relating group- and subgroup-differences to the z-maps of healthy controls, two patterns emerged for the delta- to beta-band: MS-patients had higher centrality values over the bi-parietal hub areas and lower centrality values over both temporal regions, which had medium high centrality values in healthy controls. In the gamma-band the pattern was different: MS-patients had lower centrality values over bi-parietal sensors.

### Associations between Eigenvector Centrality and Cognition

Figures 1d, 2d and S4 show the distribution of associations between EC-values to the cognition-score as r-maps and corresponding p-values in healthy controls, Figures 1e, 2e and S5 in patients. Table 3 gives the number of significantly correlated sensors per region and frequency band for the patient group. In healthy subjects (Figures 1d, 2d and S4a–f), the main cluster of positive correlations shifted from bi-frontal sensors (delta-band) mainly over left fronto-temporal sensors (theta- to upper alpha-band) to bi-centro-parietal sensors (beta- and gamma-band); however, at only four sensors in three different frequency bands (theta-, lower alpha- and gamma-band) did correlations reach statistical significance (0.51<r<0.59 and −0.52<r<−0.51, p<0.05 corrected). In patients (Figures 1e, 2e and S5a–f), the main difference to the patterns observed in healthy controls was found in temporal sensors: there were strong correlations between EC and cognitive performance (0.44<r<0.6; theta- to beta-band; p<0.01), highest over left temporal (lower alpha-band) and right temporal sensors (beta-band) (p<0.05, corrected; Figure S5c and e). Three right parieto-temporal sensors (beta-band) had a significant negative correlation to cognition (−0.5<r<−0.45, p<0.01 and p<0.05 corrected).

**Table 3:** Relationship between nodal centrality and “cognition”: number of significantly correlated sensors per region, side and frequency band in MS patients.

	frontal		temporal		Central		Parietal		occipital	
band	left	right	left	right	left	right	left	Right	left	right
delta	–	–	–	–	–	–	–	–	–	–
theta	–	–	2	1	–	–	–	–	1	–
lower alpha	1	–	9(7)	4(1)	–	–	–	–	1(1)	1
upper alpha	–	–	–	2	–	–	–	–	–	–
beta	–	1(1)	–	15(11)	–	–	–	1(1)	–	–
gamma	–	–	–	–	–	–	–	–	–	–

Number of sensors with significant correlations (p<0.01; in brackets: p<0.05 corrected); for definition of frequency bands refer to Table 2.

Relating these findings to the topography of nodal centrality distribution in healthy controls and the topography of differences between the groups, point to the temporal regions as being the most affected and associated with cognitive functioning: temporal nodal centrality values, which were in the medium range in healthy controls, were lower in MS-patients but highly positively correlated to cognitive performance.

## Discussion

To gain deeper insight into possible mechanisms underlying cognitive dysfunction in MS, we characterized the relationship of cognitive performance to patterns of altered resting-state centrality. We used the synchronization likelihood (SL) to determine functional connectivity between MEG-sensors, and the resulting weight matrix to compute the Eigenvector Centrality (EC) per sensor. EC quantifies how central a node is by accounting for its connectivity and importance within the entire network. In MS patients, nodal centrality was decreased over left temporal (upper alpha- and beta-band) and increased over bi-parietal regions (theta-band); cognitive dysfunction was correlated to lower nodal centrality over temporal regions (lower alpha- and beta-band) and higher nodal centrality over the right parietal region (beta-band). Differences were most pronounced in the male subgroup.

Earlier MEG- and EEG-studies have reported decreased inter-hemispheric, mostly inter-temporal, connectivity in the alpha-band [30], as well as decreased inter-hemispheric (theta- and alpha-band) and intra-hemispheric (alpha-band) connectivity, which was most pronounced in cognitively impaired patients [29]. Based on SL, which quantifies connectivity between two given regions, we found in our previous analysis decreased connectivity only in the upper alpha-band, and increased connectivity, mostly involving parieto-occipital regions bilaterally in several frequency bands (theta-, lower alpha-, beta-band) [20]. In the present study decreased nodal centrality over both temporal regions (upper alpha- and beta-band) was the main finding and more pronounced than shown with regional SL analysis. As EC accounts for connectivity and importance within the whole network and is a vector-normalized measure, it seems to be more sensitive to change than SL. However, the patterns found with SL and EC were consistent: the topographies of EC-changes over the different frequency bands showed also increased nodal centrality over posterior regions.

Decreased nodal connectivity over temporal regions was associated with lower cognitive performance, and whole brain grey matter atrophy was more pronounced in the cognitively more affected male patients. These observations suggest that partial functional disconnection of temporal regions is probably an important factor for cognitive dysfunction in early MS and possibly associated with structural changes and gender. Although from our current analysis we cannot make inferences on the relationship between localization of structural changes and damaged network regions, it is intriguing that an association between decreased resting-state connectivity of the hippocampi and hippocampal atrophy has recently been shown [47], and that fronto-temporal regions seem to be a predilection site of grey matter atrophy which is related to cognitive function [48]–[51]. However, changes in functional connectivity may be remote to structural lesions when nodes that connect brain regions are affected [15].

In healthy controls, the spatial distribution of nodal centrality was quite similar from the theta- to gamma-band, an observation which has been described previously [52]. Highest nodal centrality was found over parietal regions, most consistently in the upper alpha to gamma-frequency range identifying these areas as hubs. Intriguingly, parietal multi-sensory association areas have been shown with MRI to be the main structural hubs [53], and they form an important part of the default mode network (DMN) [54]. The relationship between brain oscillations and resting state networks (RSN) is complex [55]–[56]. RSN involved in higher cognitive functioning as the DMN, fronto-parietal control, frontal attention and working memory network have been found to be preferentially associated with fluctuations in the alpha- and beta-range over posterior regions [57]–[58], the DMN especially with parietal beta [58]. Thus we speculate that parietal hubs partly reflect similar substrates as the RSN and possibly the DMN.

Interestingly, alterations of RSN, particularly the DMN have been described recently in different phases of MS and showed differences between cognitively preserved and impaired patients [59]–[61]. Moreover, the negative relationship between cognitive abilities and increased functional connectivity in the DMN as well as the attention and cognitive control network has been hypothesized to speak for maladaption rather than compensation [8]. Our finding of increased nodal centrality over the (right) parietal regions associated with lower cognitive performance may be interpreted as pointing in the same direction making maladaption more likely than compensation. From a network perspective we speculate that decreased centrality in damaged regions leads to a shift of information flow to intact parts of the network strengthening their centrality, but not necessarily implying higher effectiveness. Whether the lateralization to the right parietal region has in itself relevance remains unclear but could possibly be related to some bias to right hemispheric functions in the cognition score. However, the hypothesis of a network shift has to be studied in more detail also considering the possibility that connectivity and subsequent centrality changes may be transient and dependent on disease stage and extent of cognitive impairment. Interestingly, increased functional connectivity as measured with SL was also reported in glioma patients suffering from epilepsy [19], and in Parkinson’s disease in early (lower alpha-band) and moderate advanced disease (theta, lower alpha and beta-band) [18].

The observation of a clear gender difference in our early inception cohort with quite homogenous disease duration needs to be confirmed in independent studies and bigger samples. However, sex-specific differences in the immune and nervous system have been reported previously as important factors in MS [62]. Furthermore, it has been shown that women have a greater overall cortical connectivity and a more efficient global and local network organisation as studied with MRI-tractography [63], as well as higher local functional connectivity as studied with fMRI [64], possibly rendering female brains more resilient to cognitive dysfunction.

Eigenvector centrality was used to quantify connectivity and importance within the whole network at the level of individual nodes allowing exploration of sensor-level based patterns without *a priori* assumptions on spatial distribution. Spatial imprecision due to varying head position between subjects during MEG recordings (usually within a range of a few centimetres) and consecutive smearing of the signal at the group level is counter-balanced by the fact that neighbouring sensors are highly inter-correlated anyway due to field spread [65] and volume conduction. These factors may influence the SL-based estimates of functional connectivity to some extent. However, as there is no obvious reason to assume that field spread and volume conduction differ between MS patients and healthy controls, our reported group differences are most likely due to underlying pathological changes in MS. Importantly, in our previous analysis of the same data the main findings were unaffected when a connectivity measure was used that is insensitive to the effects of volume conduction (imaginary phase coherence [66]).

The strength of a node, which is in binary networks its degree centrality, can be determined by averaging the connectivity values over all its connections that a given node has with any other node in the network. Compared to degree centrality, EC has two main advantages: it “weighs” the connectivity by also taking the connectivity of the neighbouring nodes into account, thus determining the importance of a node within the entire network, and it is a vector-normalized relative measure, facilitating comparisons between equal sized networks. EC has been shown to be an effective measure for model free analysis of large datasets [22] and suited to detect hubs at a global level [23]–[24]; however, it is less sensitive to detect hubs in modules after partitioning the network [23]. In our case of small networks, we consider EC an appropriate choice as the main goal was to define the major hub and to provide between-network comparability.

Statistical power had to be adjusted to a mainly explorative level (p<0.01 uncorrected), probably due to the relatively small sample size and the heterogeneity in expression of cognitive symptoms in our sample, which ranged from normal to subtle dysfunction to mild impairment in eight patients. However, sensors significant at the chosen threshold were mainly found in clusters of at least three adjacent sensors, making chance findings unlikely.

### Conclusion

The present study showed that nodal centrality in resting-state MEG is altered in the early phase of MS, and more pronounced in male patients, who also had more cognitive dysfunction. The topography of group- and subgroup differences showed a dichotomous pattern with partial disconnection of temporal regions and increased centrality in parietal hubs. Cognitive dysfunction was related to partial temporal disconnection and, to a much lesser extent to increased parietal centrality; the latter finding may indicate dysfunctional network rearrangements rather than adaptive compensation. Longitudinal studies are needed to further elucidate the relationship between structural, functional and cognitive changes, as well as gender effects in MS. MEG derived analysis of resting-state functional connectivity using Eigenvector Centrality is a useful tool for this purpose.

## Supporting Information

Figure S1 Spatial distribution of z-transformed group averaged EC-values per sensor in healthy controls over the six frequency bands plotted as z-maps: a) delta- (0.5–4.0 Hz), b) theta- (4.0–8.0 Hz), c) lower alpha- (8.0–10.0 Hz), d) upper alpha- (10.0–13.0), e) beta- (13.0–30.0 Hz) and f) gamma-band (30.0–48.0 Hz); g)–l): corresponding distribution of sensors, which belong in > = 50% and > = 80% of healthy subjects to the 20% highest EC-values of each individual subject (group-“hubness”).
(TIF)

Figure S2 Spatial distribution of group-differences plotted as a t-map over the six frequency bands a)–f) and corresponding p-values g)–l); warm colors indicate higher values in MS.
(TIF)

Figure S3 Spatial distribution of differences in the subgroup of men plotted as a t-map over the six frequency bands a)–f) and corresponding p-values g)–l); warm colors indicate higher values in MS.
(TIF)

Figure S4 Spatial distribution of correlations between “cognition” and EC-value per sensor in healthy controls plotted as r-maps over the six frequency bands a)–f) and corresponding p-values g)–l): warm colors indicate positive correlation.
(TIF)

Figure S5 Spatial distribution of correlations between “cognition” and EC-value per sensor in MS-patients plotted as r-maps over the six frequency bands a)–f) and corresponding p-values g)–l): warm colors indicate positive correlation.
(TIF)
