# Supplementary figures and images for: Cognitive Dysfunction in Early Multiple Sclerosis: Altered Centrality Derived from Resting-State Functional Connectivity Using Magneto-Encephalography
Source: PLoS One. 2012 Jul 27;7(7):e42087. doi: 10.1371/journal.pone.0042087 (PMC3407108; doi:10.1371/journal.pone.0042087)

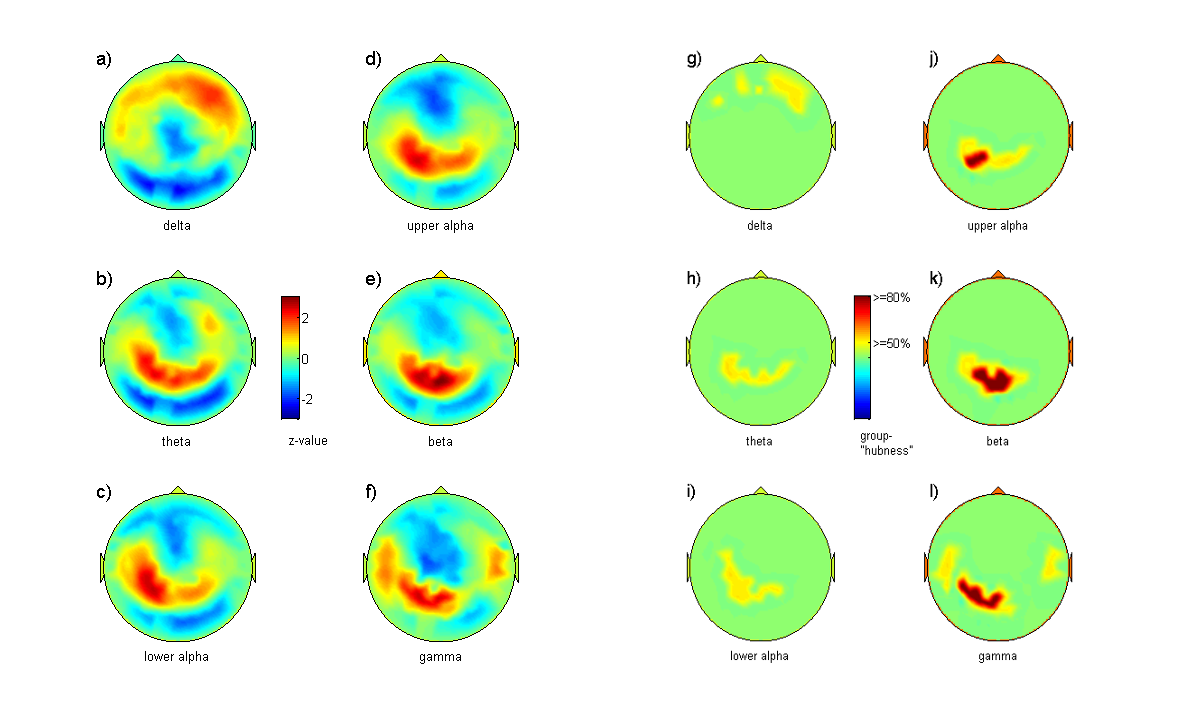

Supplement: Figure S1 — Spatial distribution of z-transformed group averaged EC-values per sensor in healthy controls over the six frequency bands plotted as z-maps: a) delta- (0.5–4.0 Hz), b) theta- (4.0–8.0 Hz), c) lower alpha- (8.0–10.0 Hz), d) upper alpha- (10.0–13.0), e) beta- (13.0–30.0 Hz) and f) gamma-band (30.0–48.0 Hz); g)–l): corresponding distribution of sensors, which belong in > = 50% and > = 80% of healthy subjects to the 20% highest EC-values of each individual subject (group-“hubness”). (TIF) [file pone.0042087.s001.tif]

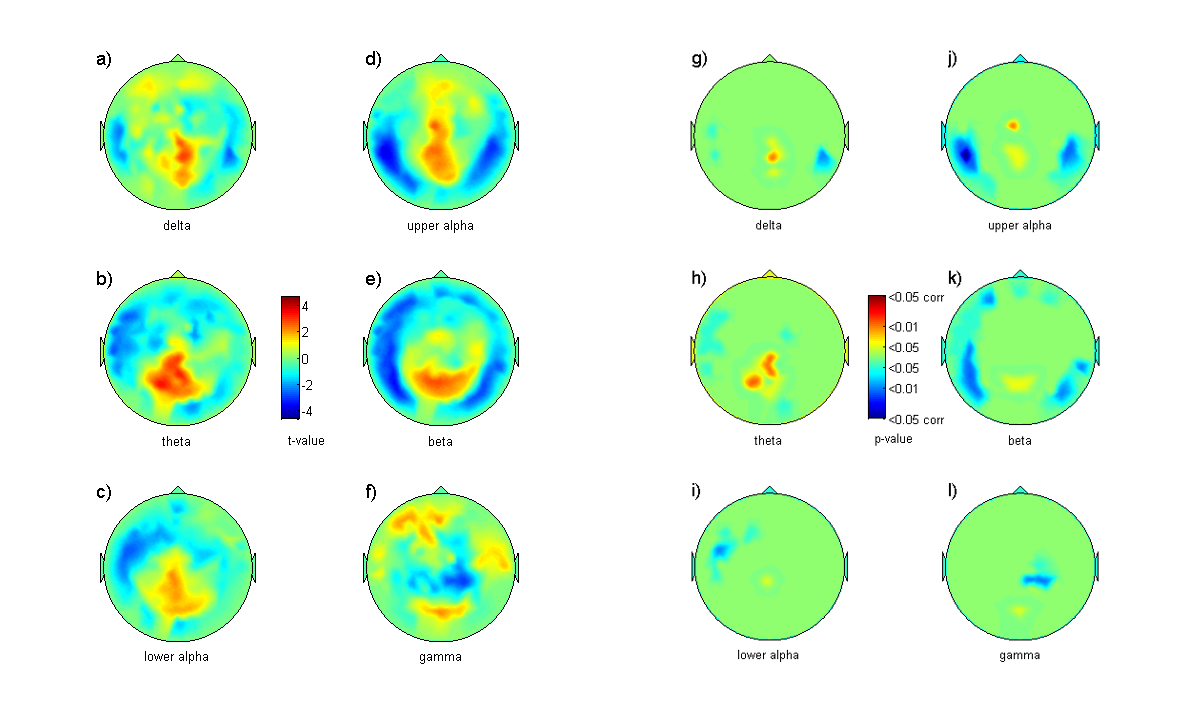

Supplement: Figure S2 — Spatial distribution of group-differences plotted as a t-map over the six frequency bands a)–f) and corresponding p-values g)–l); warm colors indicate higher values in MS. (TIF) [file pone.0042087.s002.tif]

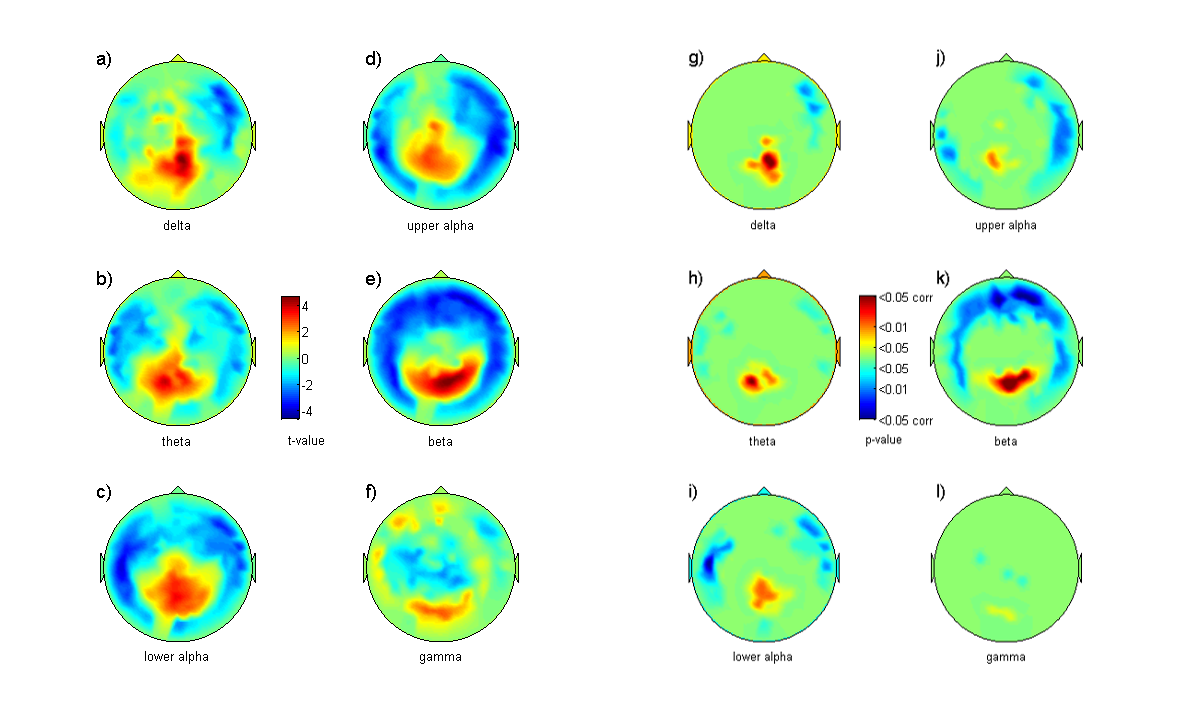

Supplement: Figure S3 — Spatial distribution of differences in the subgroup of men plotted as a t-map over the six frequency bands a)–f) and corresponding p-values g)–l); warm colors indicate higher values in MS. (TIF) [file pone.0042087.s003.tif]

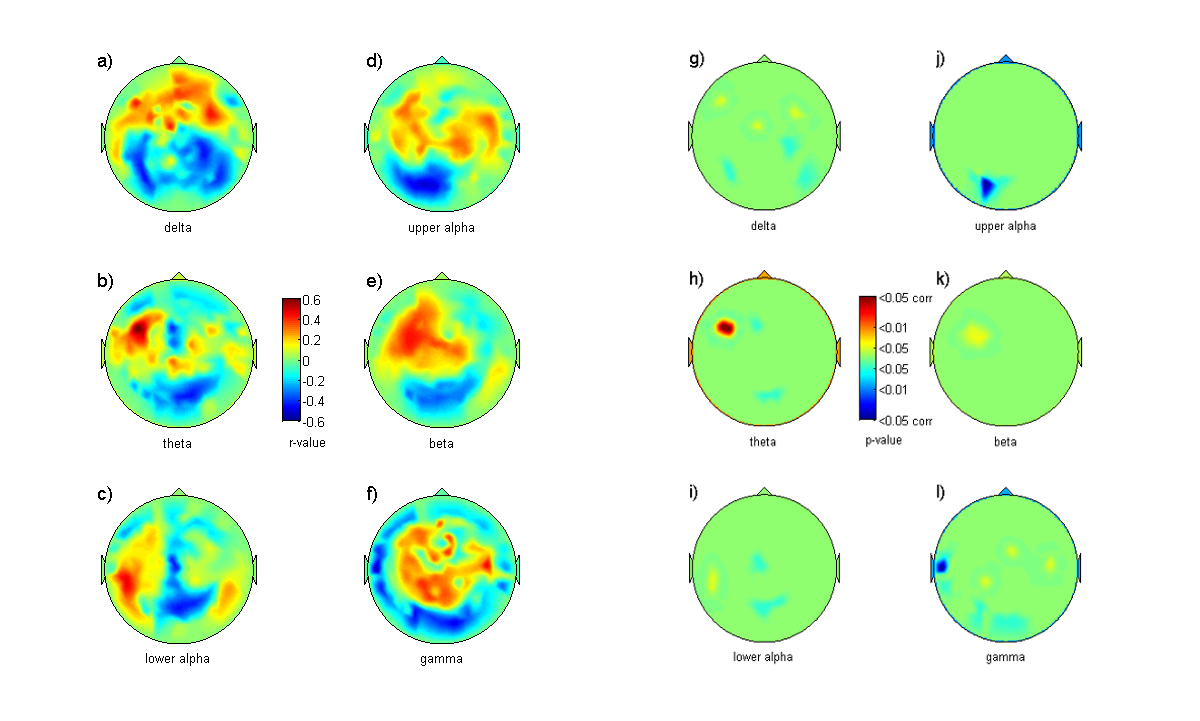

Supplement: Figure S4 — Spatial distribution of correlations between “cognition” and EC-value per sensor in healthy controls plotted as r-maps over the six frequency bands a)–f) and corresponding p-values g)–l): warm colors indicate positive correlation. (TIF) [file pone.0042087.s004.tif]

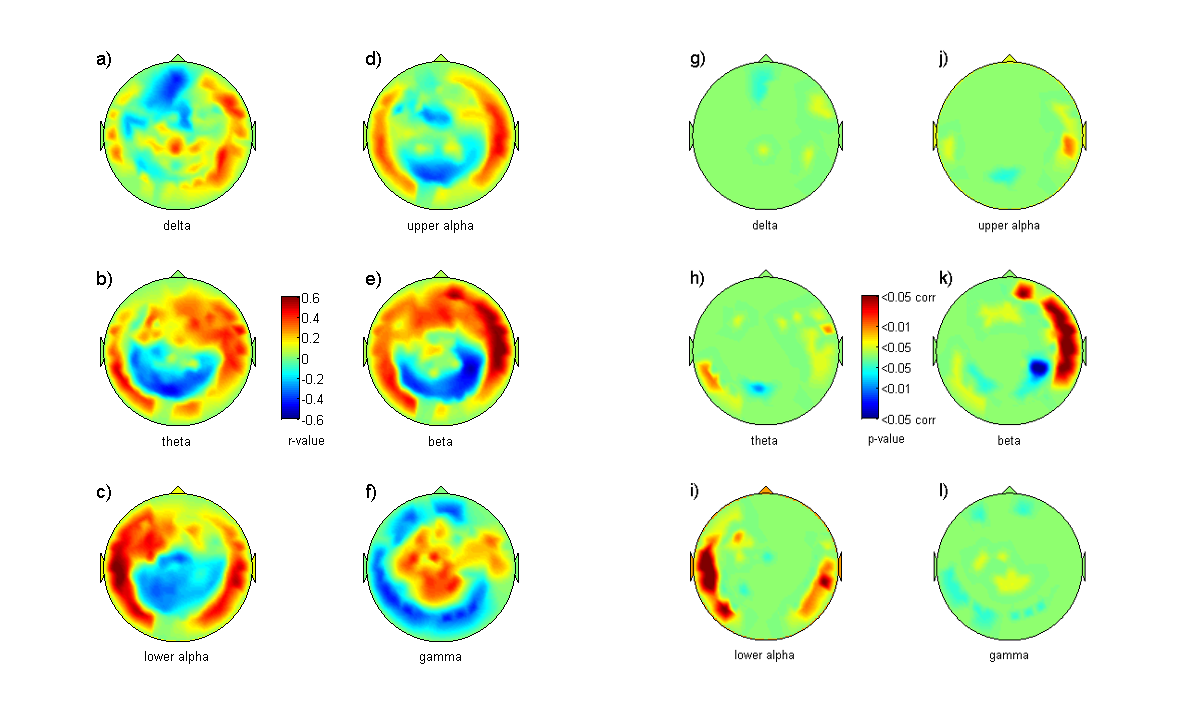

Supplement: Figure S5 — Spatial distribution of correlations between “cognition” and EC-value per sensor in MS-patients plotted as r-maps over the six frequency bands a)–f) and corresponding p-values g)–l): warm colors indicate positive correlation. (TIF) [file pone.0042087.s005.tif]
